# Technical Workflow Development for Integrating Drone Surveys and Entomological Sampling to Characterise Aquatic Larval Habitats of *Anopheles funestus* in Agricultural Landscapes in Côte d'Ivoire

**DOI:** 10.1155/2021/3220244

**Published:** 2021-11-01

**Authors:** Isabel Byrne, Kallista Chan, Edgar Manrique, Jo Lines, Rosine Z. Wolie, Fedra Trujillano, Gabriel Jimenez Garay, Miguel Nunez Del Prado Cortez, Hugo Alatrista-Salas, Eleanore Sternberg, Jackie Cook, Raphael N'Guessan, Alphonsine Koffi, Ludovic P Ahoua Alou, Nombre Apollinaire, Louisa A. Messenger, Mojca Kristan, Gabriel Carrasco-Escobar, Kimberly Fornace

**Affiliations:** ^1^Department of Disease Control, London School of Hygiene & Tropical Medicine, London WC1E 7HT, UK; ^2^Centre on Climate Change and Planetary Health, London School of Hygiene & Tropical Medicine, London WC1E 7HT, UK; ^3^Health Innovation Laboratory, Institute of Tropical Medicine “Alexander von Humboldt”, Universidad Peruana Cayetano Heredia, Lima, Peru; ^4^Laboratorio ICEMR-Amazonia, Laboratorios de Investigación y Desarrollo, Facultad de Ciencias y Filosofía, Universidad Peruana Cayetano Heredia, Lima, Peru; ^5^Institut Pierre Richet, Bouaké, Côte d'Ivoire; ^6^Laboratoire de génétique, Unité de Formation et de Recherche en Biosciences, Université Félix Houphouët Boigny, Abidjan, Côte d'Ivoire; ^7^Universidad Peruana Cayetano Heredia, Lima, Peru; ^8^Universidad de Ingenieria y Tecnología, Lima, Peru; ^9^Pontificia Universidad Católica Del Perú, Lima, Peru; ^10^Department of Vector Biology, Liverpool School of Tropical Medicine, Liverpool, UK; ^11^Department of Infectious Disease Epidemiology, London School of Hygiene & Tropical Medicine, London WC1E 7HT, UK; ^12^University of Ouagadougou, Ouagadougou, Burkina Faso; ^13^Scripps Institution of Oceanography, University of California San Diego, La Jolla, CA, USA; ^14^Institute of Biodiversity, Animal Health & Comparative Medicine, University of Glasgow, Glasgow, UK

## Abstract

Land-use practices such as agriculture can impact mosquito vector breeding ecology, resulting in changes in disease transmission. The typical breeding habitats of Africa's second most important malaria vector *Anopheles funestus* are large, semipermanent water bodies, which make them potential candidates for targeted larval source management. This is a technical workflow for the integration of drone surveys and mosquito larval sampling, designed for a case study aiming to characterise *An. funestus* breeding sites near two villages in an agricultural setting in Côte d'Ivoire. Using satellite remote sensing data, we developed an environmentally and spatially representative sampling frame and conducted paired mosquito larvae and drone mapping surveys from June to August 2021. To categorise the drone imagery, we also developed a land cover classification scheme with classes relative to *An. funestus* breeding ecology. We sampled 189 potential breeding habitats, of which 119 (63%) were positive for the *Anopheles* genus and nine (4.8%) were positive for *An. funestus*. We mapped 30.42 km^2^ of the region of interest including all water bodies which were sampled for larvae. These data can be used to inform targeted vector control efforts, although its generalisability over a large region is limited by the fine-scale nature of this study area. This paper develops protocols for integrating drone surveys and statistically rigorous entomological sampling, which can be adjusted to collect data on vector breeding habitats in other ecological contexts. Further research using data collected in this study can enable the development of deep-learning algorithms for identifying *An. funestus* breeding habitats across rural agricultural landscapes in Côte d'Ivoire and the analysis of risk factors for these sites.

## 1. Introduction

Land-use and land cover changes transform landscapes, with complex effects on mosquito vectors and the transmission of malaria and other vector-borne diseases [1, 2]. These environmental changes can lead to shifts in vector densities, species compositions, host availability, and biting patterns [3]. Despite large reductions in malaria burden globally, these landscape changes threaten to undermine malaria control and elimination efforts and have been identified as a priority for malaria eradication by the World Health Organisation [4]. Land-use practices, such as agriculture, forestry, and urbanisation, can modify physical surfaces and create new malaria vector breeding sites. Identifying where and when land-use practices are likely to increase mosquito vector densities and malaria transmission is a fundamental step towards understanding how landscapes can be sustainably managed to improve public health.

Remote sensing data is becoming increasingly accessible as a means to investigate the impact of land-use practices and other landscape characteristics on different aspects of vector ecology [5]. These Earth Observation (EO) data have different spatial and temporal resolutions depending on the sensor specifications and frequencies of obtaining usable imagery. In addition, optical EO data are characterised by spectral resolution determined by the number and wavelengths the sensor measures along the electromagnetic spectrum. Traditionally, pixel-based machine learning methods have been used to identify land cover categories of interest from these spectral signatures. However, new sources of Earth Observation data, such as very high-resolution satellite imagery (e.g., Geo-Eye and Planet SkySat) and drone imagery, often have high spatial resolution but much lower spectral resolution. Aerial imagery may also be used to estimate 3-dimensional structures using photogrammetric (structure from motion) methods [6]. While these new data sources have enormous potential for identifying vector habitats, the characteristics of these data limit the utility of pixel-based methods relying solely on spectral characteristics to classify environments. Deep-learning methods, such as convolutional neural networks (CNNs), are revolutionising image analysis. These methods allow efficient analysis of image textures and spectral characteristics using self-learning artificial intelligence approaches to identify features in complex environments. By enabling the identification of highly discriminative features and patterns, these methods have the potential to detect landscape factors associated with vector habitat productivity, helping to overcome the limitations of lower spectral resolution data. Although computationally intensive, more efficient deep-learning architectures and cloud-based computing are increasingly accessible and have been applied for ecological analysis (e.g., [7–9]).

Mosquitoes have four main life stages. The first three juvenile stages, egg, larva, and pupa, are aquatic, with the adult stage being terrestrial. When aiming to identify aquatic mosquito habitats, the choice of EO data and analysis methods is largely dependent on the local vector ecology and available resources. *Anopheles funestus*, the second most important malaria vector in Africa, typically breeds in large permanent or semipermanent water bodies with emergent vegetation [10–13]. The creation of these habitats has been associated with agricultural practices such as rice cultivation [14–16], pastures [17], cultivated swamps [18–20], and canals and drainage ditches used for irrigation [10–12, 21, 22]. The relatively large and stable qualities of *An. funestus* habitats suggest the utility of satellite-based EO data to identify breeding sites despite coarser spatial resolution and less frequent coverage of satellites over target areas. The identification of breeding sites can allow for targeted malaria control using larval source management, or LSM. The goal of LSM is to reduce adult mosquito populations by targeting the immature aquatic stages of disease vectors through environmental, biological, or chemical modification of the larval habitats [23]. Using LSM in areas where this vector dominates has been suggested as a potential tool to complement existing malaria control efforts due to the “few, fixed, and findable” nature of their breeding sites [13]. As satellite-based EO data are often available freely through government sources (e.g., NASA and the European Space Agency), these data sources may be more attractive for control programmes operating in resource-limited environments. However, although satellite-based EO data have been used to characterise risk factors for *An. funestus* breeding sites (e.g., [24, 25]) and describe *An. funestus* species distributions (e.g., [26]), predictive models integrating EO data have not been routinely applied to identify *An. funestus* breeding sites as targets for vector control. A previous study found the pixel-based analysis of spectral signatures of EO data were insufficient to identify *An. funestus* breeding sites [27]. Subsequent studies have visually identified these breeding habitats from very high-resolution aerial imagery collected by drones [28]. In comparison to satellite-based EO data, aerial drone surveys are more resource intensive and require field-based teams, but they may generate data that are more easily interpretable and actionable for local control programmes.

Despite the medical importance of this vector, and the nature of breeding water bodies which intuitively should be relatively easy to detect, *An. funestus* breeding habitats are difficult to locate [13]; however, there is a paucity of knowledge on their breeding ecology in Côte d'Ivoire. Developing operationally feasible methods of identifying potential breeding sites for *An. funestus* requires integrating different sources of EO data with local entomological knowledge. Baseline knowledge of the spatial distribution and characteristics of their larval habitats are required and can be acquired by conducting larval surveys during the appropriate months. An important requirement in the collection of larval distribution data is to ensure that all habitats present in the study area are sampled equally. Ensuring such spatial representativeness can help to build an understanding of the distribution of breeding sites across the full range of land types and habitats present in the study site, rather than an overrepresentation of habitats which are easy to access or are suspected to be associated with larval presence [29, 30].

One method of ensuring environmental and spatial representativeness in a larval sampling frame is by classifying the region of interest into environmental strata such as land cover and randomly sampling each stratum proportionally. This ensures a random spatial distribution of sampled sites, with an even number of sampled sites for each environmental stratum. EO data such as drone imagery and multispectral satellite data have been used to develop stratified sampling designs for malaria vector larval surveys [29, 31]. In the case of *An. funestus* breeding habitats and their documented rarity [13], a representative sampling design can be integrated with local entomological and hydrological knowledge. This should ensure that the aquatic habitats which could potentially serve as sites for *An. funestus* oviposition within the study site are located. The locations of potential *An. funestus* breeding sites identified through the representative sampling frame can then be used to guide a second stage of intensive larval surveys. Such adaptive sampling designs, which use information from previously collected data to inform future sampling sites, have been used to aid the field studies in locating rare or hard-to-find phenomena such as plant species or disease hotspots [32–34].

While ground-based studies and aerial mapping may be essential for identifying fine-scale characteristics of water bodies and determining vector productivity, regional mapping of large areas can only be achieved by using satellite-based EO data. The utility of these different data sources and image analysis methods are highly dependent on the identification of fine-scale landscape factors influencing vector distributions. Deep learning methods of image classification are highly reliant on the availability of sufficient training data on areas with and without vector breeding sites. This necessitates designing a spatially representative sampling approach to identify breeding sites and key landscape features across areas of interest. The aim of this study was to characterise the aquatic habitats of *An. funestus* across an agricultural gradient in Côte d'Ivoire using paired drone and entomological surveys. In this paper, we outline a technical workflow to carry out these aims and to collect data to enable development of deep-learning approaches to identify *An. funestus* breeding sites. The specific objectives were to (i) develop a land cover classification scheme with categories relative to *An. funestus* breeding and to categorise drone images accordingly; (ii) to develop a statistically rigorous larval sampling frame for *An. funestus* which is environmentally and spatially representative of the study site using remote sensing data and which is spatially adaptive to account for the rarity of breeding sites; and (iii) to conduct paired larval and high resolution drone surveys and collect independent validation data for the land classification scheme.

## 2. Methods

### 2.1. Study Area

This study was conducted in two villages southeast of Bouaké in the Gbêkê region of the Vallée du Bandama district of central Côte d'Ivoire. In this region, malaria is endemic with year-round transmission peaking during the rainy season between May and October [35]; whilst the dominant malaria vector species is *An. gambiae* sensu lato, *An. funestus* s.l., *An. nili,* and *An. ziemanni* are also present. The average annual rainfall is 1200 mm, and there is an average annual temperature of 25.8°C [14].

### 2.2. Study Design and Site Selection

**Figure 1:**
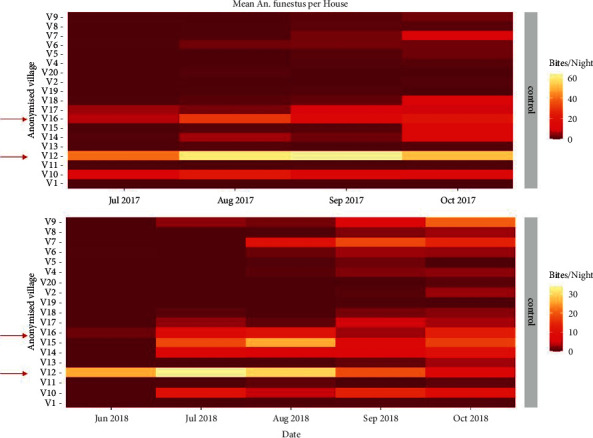
The mean *An. funestus* count per house in control villages from the study [35] from June to September 2017 and 2018. Our study villages, chosen for the highest *An. funestus* count during the season of interest, are highlighted with red arrows.

**Figure 2:**
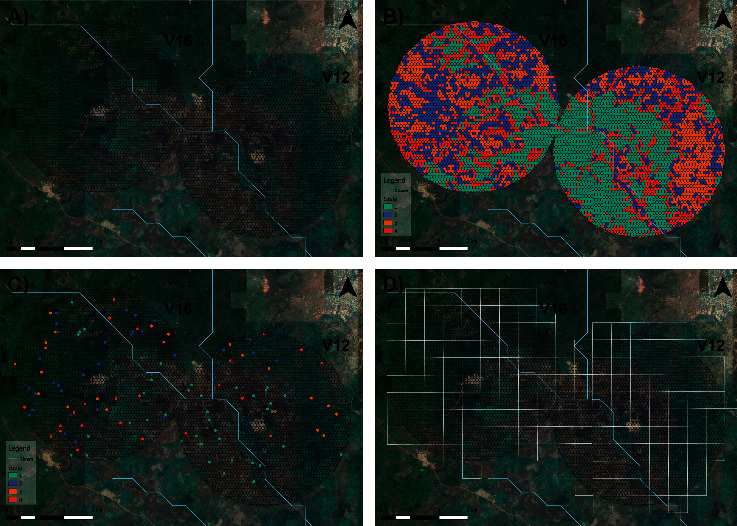
Three km buffer denoting the region of interest around two study villages, divided into (a) one-hectare hexagons, which were used as sampling units for the larval survey, and (b) stratified based on their multispectral and hydrological profiles; (c) hexagons selected for sampling from the randomly stratified sampling strategy; and (d) 650 m × 600 m grid guidelines for drone surveys.

This was a cross-sectional study pairing larval surveys of *An. funestus* with drone surveys to capture the landscape composition that drives the fine-scale spatial distribution of *An. funestus* breeding sites in two villages in central Côte d'Ivoire. The two villages were chosen based on adult mosquito catch data collected during a randomised controlled trial on the impact of structural interventions on malaria transmission from 2017 to 2019 [35]. Specifically, “control” villages (i.e., without structural interventions) with high mean nightly catches of *An. funestus* (using human landing catches and CDC light traps) as well as high proportions of *An. funestus* compared to overall *Anopheles* numbers were selected (Figure 1). The region of interest was set as a 3 km circular buffer (12.6 km^2^) surrounding the centroid of each village (Figure 2), corresponding to the average mosquito flight range [36].

### 2.3. Land Classification Development

#### 2.3.1. Defining Land Classes of Interest

We reviewed the scientific literature for descriptions of land cover types and water body characteristics which are associated with *An. funestus* larval and adult stages. This informed the development of a hierarchical land classification scheme for the Côte d'Ivoire study site. An initial scoping assessment of freely available high-resolution satellite data (https://earth.google.com/web/and https://www.planet.com/explorer) was performed. This assessment allowed us to determine which of the land classes and water body types identified from the literature review were present in the Côte d'Ivoire study sites and were discernible from high-resolution remote sensing data. The presence of these land classes and water body types were subsequently validated by field staff who were familiar with the landscape. The resulting land classification scheme was used in ground truthing field surveys.

### 2.4. Sampling Frame

Two stages of sampling were used to locate potential *An. funestus* larval habitats. The first was a randomly stratified sampling frame with targeted sampling, where the landscape was divided into environmental strata using remote sensing data, and each stratum was sampled proportionally. The second sampling stage was an adaptive sampling strategy to account for the low predicted number of *An. funestus* breeding sites within the study area.

#### 2.4.1. Randomly Stratified and Targeted Sampling Stage

A hexagonal grid was used to partition the region of interest into sampling units of 10,000 m^2^. Hexagons were used because their centroids are equidistant to the perimeter of each edge. Sentinel-2 MSI: MultiSpectral Instrument, Level-2A, wide-swath high-resolution multispectral imagery was retrieved from Google Earth Engine (GEE). The imagery was gathered from 1st January 2019 to 31st December 2020, all with less than 20% cloud coverage. The imagery was used to compute the following vegetation indices: normalised difference vegetation index (NDVI), normalised difference water index (NDWI), enhanced vegetation index (EVI), and soil adjusted vegetation index (SAVI). The GEE script with the preprocessing of the Sentinel-2 imagery and computation of the vegetation indices is provided in Supplementary Materials 1. The equations used to compute the vegetation indices are as follows:

(1) $$\begin{array}{c} \text{NDVI}=\frac{\left(\text{NIR }-\text{Red}\right)}{\left(\text{NIR}+\text{Red}\right)},\\ \text{NDWI}=\;\frac{\left(\text{NIR}-\text{ SWIR}\right)}{\left(\text{NIR}+\text{SWIR}\right)},\\ \text{EVI}=\;2.5\;\times \frac{\left(\text{NIR}-\text{Red}\right)}{\left(\text{NIR}+\left(6\;\times \text{ Red}\right)\;-\left(7.5\;\times \text{ Blue}\right)+1\right)},\\ \text{SAVI}=1.5\;\times \frac{\left(\text{NIR}-\text{Red}\right)}{\left(\text{NIR}+\text{Red}+0.5\right)}. \end{array}$$

A k-means clustering algorithm was then applied to group hexagons into four environmental strata based on their multispectral and environmental profiles (Figure 2(a)).

**Figure 3:**
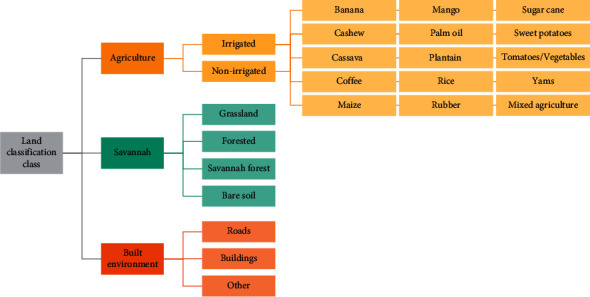
Land classification scheme for field-based ground truthing.

Based on the average time taken to sample a hexagon for a team of three fieldworkers, a sample size of 70 hexagons per village was chosen for this initial sampling stage. The 70 hexagons were randomly selected proportional to the total number of sampling units of each stratum across the region of interest (Figure 2(b)). They were then assigned a unique identifier code based on the village, stratum, and position within the area of interest. To locate the randomly selected sampling unit within the field and to ensure that sampling was carried out within the boundaries of the hexagon, an offline satellite map overlaid with the hexagons of interest was created using QGIS v3.12 [37] as an MBTile. The maps were uploaded to the OpenDataKit (ODK) software [38] onto Android tablets, and the GPS coordinates of the tablet were visualised on the offline map using the geotrace function. Sampling one hexagon consisted of collecting three ground truthing points and sampling all potential larval habitats for larvae. At each ground truthing point, the GPS coordinates and photos were collected using digital forms installed in tablets through ODK, and the predominant land cover type was recorded according to the land cover classification scheme, which captures agricultural, savannah, and built environments (Figure 3).

Due to the scarceness and documented difficulty in finding *An. funestus* breeding sites [13], we conducted targeted sampling alongside the stratified sampling by actively searching for water bodies in the vicinity of the randomly stratified hexagons. These surveys were aided by two members of the village, who are very familiar with the locations of water bodies and their seasonality. The geotrace function on the ODK software was also used to record the GPS coordinates of the hexagons which were visited during the targeted surveys.

#### 2.4.2. Adaptive Sampling Stage

Using the GPS coordinates of the water bodies recorded during the first stage of the sampling frame, we conducted an intensive second stage of larval sampling. This involved returning to all previously identified water bodies and sampling for larvae and prospective searches within these areas to find similar water bodies nearby, aided again by the village member's knowledge.

### 2.5. Larval Survey, Environmental DNA Collection, and Water Body Characteristics

Larval surveys were conducted at every water body found using the two sampling strategies. Each sample included larval dips, an environmental DNA sample, and the collection of water body characteristics.

Larvae were sampled using standard larval dippers (BioQuip 350 mL), with five dips conducted at each sampling spot. For water bodies that had a clear distinction between areas with and without emergent vegetation, two samples were performed accordingly. Counts of *Anopheles* immatures were recorded according to their developmental stage: early (L1/L2), late instars (L3/L4), and pupae. They were transferred into QR code-labelled vials for transportation to the entomological laboratory, where they were reared into adults for morphological species identification using a dissecting microscope. The presence or absence of culicine larvae was also recorded. The standard operating procedures for larvae collections and rearing are outlined in Supplementary Materials 2A.

In order to confirm the absence or presence of *An. funestus* larvae, water samples were also collected at every sampling spot for environmental DNA (eDNA) analysis. These were collected into QR code-labelled 50 mL Falcon tubes and stored in a freezer for transportation to the entomological laboratory, where they were stored at −80°C. eDNA was extracted according to the protocol outlined in Supplementary Materials 2B. Taqman quantitative PCR assays were then used to detect eDNA from *An. funestus* with probes targeting the species-specific sequences of five *An. funestus* species available in the National Center for Biotechnology Information (NCBI) database [39].

Regardless of *Anopheles*' absence or presence, the physicochemical parameters of each water body were collected in ODK forms. The parameters of interest and the equipment used to collect them are listed in Table 1.

### 2.6. Drone Surveys

Drone surveys were carried out using a DJI Phantom 4 Pro (DJI, Shenzhen, China) quadcopter fitted with a DJI 4K camera (8.8 mm/24 mm; f/2.8; 1″ CMOS; 20 MP). Flight plans were programmed using Pix4Dcapture and DJI GS Pro mapping applications on an iPad Pro (Apple, California, US). Connections between the drones and controllers were set up using the DJI Go 4 application.

Red-green-blue (RGB) mapping was conducted in the areas surrounding the water bodies found during the larval sampling. Drones were flown at an altitude of 150 m, generating imagery with a pixel size of ∼4 cm. Photographs were taken using a “fast” picture trigger mode with a 70% forward and horizontal overlap to generate seamless image mosaics. Drones were programmed to fly 650 × 600 metres (0.40 km^2^) parcel sizes from takeoff points identified using grids overlaid on satellite imagery (Figure 2). Each flight took 15 minutes, depending on time to reach the start point of the grid.

A flight log was used to make note of the general conditions at which the drones were flown (Supplementary Materials 4). The standard operating procedure for mapping breeding sites with drones is available under Supplementary Materials 2C. Information sheets in French and the local Baoule languages on the use of drones were circulated around the sample villages (Supplementary Materials 5).

## 3. Data Analysis

### 3.1. Data Management

To ensure confidentiality, field data (larval and ground-truth surveys) were encrypted, stored on secured tablets, and sent to a private server. Drone imagery was kept in an encrypted hard drive on a password-protected computer. Morphological identification of adult *Anopheles* mosquitoes was transcribed and stored in an encrypted folder.

### 3.2. Orthomosaic Construction

Drone imagery was combined into high-resolution image mosaics through photogrammetric processing using AgiSoft Metashape Professional (https://www.agisoft.com). First, drone imagery, which is automatically recorded with GPS coordinates, is imported into MetaShape and processed to construct an orthomosaic (a georeferenced mosaic of overlapped images). Second, photographs are aligned (accuracy: high; generic and reference preselection active; key point limit: 40,000; adaptive camera model fitting active) and points are matched between overlapping images to build a sparse point cloud. Third, the sparse point cloud is used as a foundation to build a dense point cloud (quality: high; and depth filtering: moderate). Fourth, the dense point cloud is used to classify ground points. Fifth, a digital elevation model (DEM) is built (geographic projection using WGS 84 (EPSG:4326); pixel size of 14.6 cm; interpolation: extrapolated; all point classes to generate a digital surface model). Finally, an orthomosaic is built (input surface: DEM; blending mode: mosaic; pixel size of 3.6).

### 3.3. Image Classification

#### 3.3.1. Classifying Remote-Sensing Imagery

The drone data were supplemented with high-resolution (0.58 m spatial resolution, RGB spectral resolution) commercial satellite data from DigitalGlobe© WorldView2 (https://discover.digitalglobe.com/), collected in September 2019 and covered the region of Gbêkê. These remote-sensing data were classified into land cover types relevant to *An. funestus* breeding site spatial distribution, according to a refined version of the classification scheme that was developed to ensure parsimony and applicability to *An. funestus* breeding ecology. The drone images were labelled according to the simplified land classification scheme by object detection methods using the free and collaborative online image labelling tool Groundwork (https://groundwork.azavea.com). Each label was validated by local field technicians and researchers from Côte d'Ivoire who are familiar with the landscapes. Labelled data were exported as georeferenced GeoJSON files.

## 4. Results

**Figure 4:**
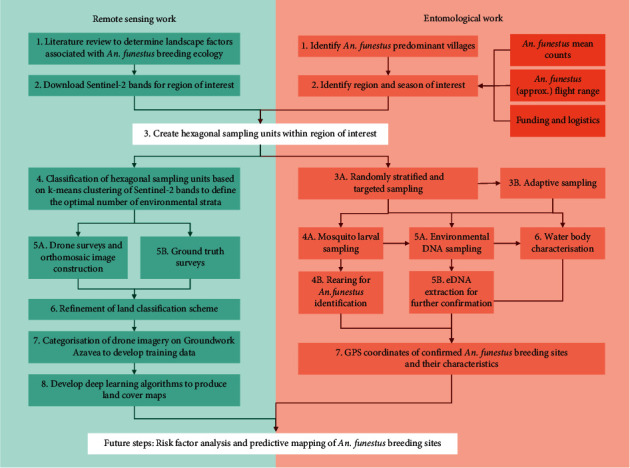
Technical workflow integrating remote sensing (including drone) and entomological work.

A summary of the technical workflow is presented in Figure 4.

### 4.1. Classification

**Figure 5:**
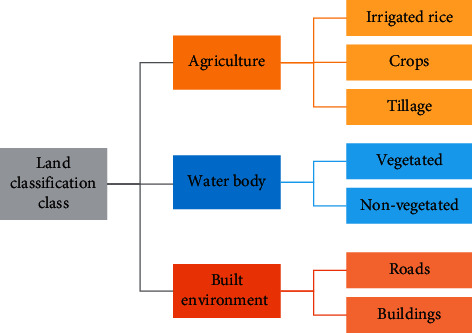
Refined land classification scheme for categorising drone imagery.

Based on a review of published literature, we identified key characteristics of potential *An. funestus* breeding sites (Table 2). These included irrigated agricultural landscapes as well as large water bodies with emergent vegetation. We additionally identified key areas in which *An. funestus* was unlikely to be detected, including roads and other built-up areas. Based on these findings and local ecology, we developed a land classification scheme specific to this area and *An. funestus* habitats (Figure 5).

### 4.2. Stratification

The optimal number of clusters from the K-means algorithm was *k* = 4, giving us 4 environmental strata which the hexagons were assigned to (Figure 2(b) and Table 3).

### 4.3. Larval Sampling

The larval and drone surveys were conducted from 24th June to 31st July 2021. The second rainy season in Côte d'Ivoire occurs from July to September. This season, however, was remarkably dry. We sampled 189 potential mosquito breeding habitats, of which 119 (63%) were positive for larvae of the *Anopheles* genus, found mostly using targeted sampling (Table 4). *Anopheles* larvae-positive habitats were more likely to be semipermanent or permanent water bodies with emergent vegetation and stationary water flow (Table 5). The anopheline species identified included *An. gambiae, An. nili, An. pharoensis*, *An. ziemanni,* and *An. funestus.* Nine sampling points (4.8%) were positive for *An. funestus* larvae. Culicine larvae were also recorded in 120 water bodies (63%) but were not counted nor speciated. eDNA samples were collected for every sampled breeding habitat. At the time of writing, the eDNA results have not been finalised.

**Figure 6:**
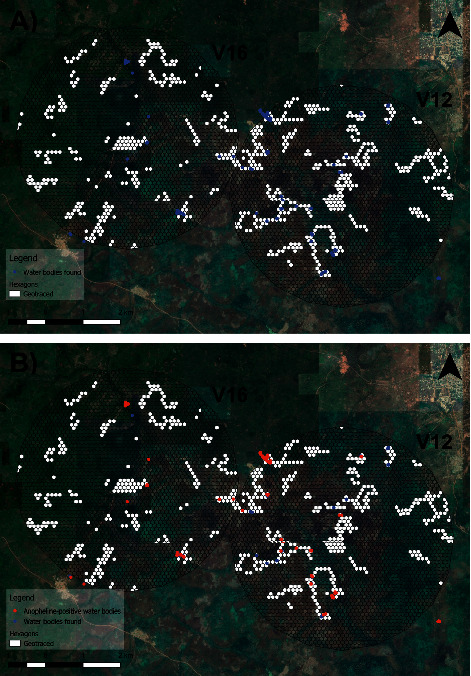
Map of (a) hexagons visited in white and water bodies in blue, of which (b) hexagons positive with *Anopheles* larvae are in red.

A total of 671 hexagons were visited during the study, 121 according to the randomly stratified sampling design and the remaining 550 were visited during targeted and adaptive surveys (Figure 6). This makes up 11.6% of the total hexagons in the full region of interest.

### 4.4. Drone Surveys

**Figure 7:**
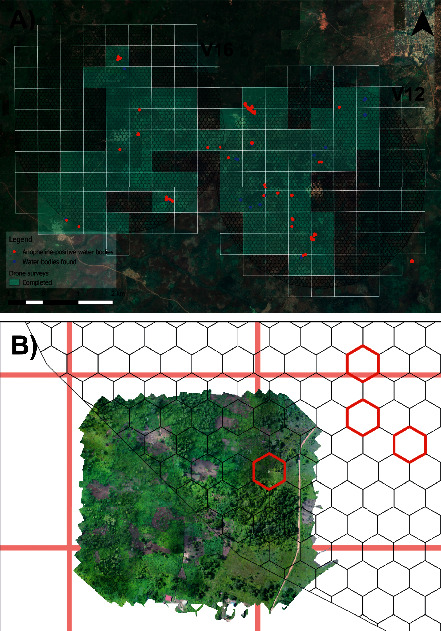
(a) A rectangular grid used to plan drone flights, with the grey coloured rectangles highlighting drone flights. (b) Example of a 600 × 650 m orthomosaic generated from a single flight. The red rectangular grid indicates the guidelines used in the field to plan the drone flights, and the red hexagons are random sampling locations.

We planned on performing 176 drone flights of 650 m × 600 m to cover the study area of 68.64 km^2^, but due to technical difficulties with the drones we were able to complete 78 flights, covering 30.42 km^2^ (Figure 7(a)). The rectangular grid was used as a guideline for the drone flights, although there could be a gap between the grid and the orthomosaic generated by a single drone flight (Figure 7(b)).

### 4.5. Ground-Truthing and Image Classification

**Figure 8:**
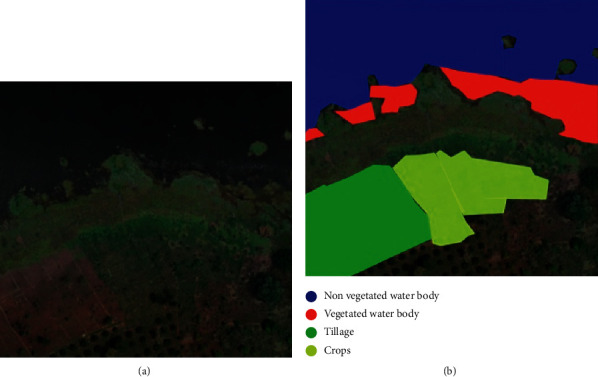
An example of a drone image categorised into four of the land classes from the refined land classification system.

From the available drone imagery and ground-based observations, we developed a workflow to manually classify different land cover categories of interest. Researchers used online tools to manually digitise the extent of different land cover categories (Figure 8).

## 5. Discussion

Within this study, we developed a systematic approach to collect and label paired entomological and aerial EO data for the development of deep-learning approaches. While drones are increasingly accessible to vector control programmes, critical gaps remain on how to design data collection and analysis methods. This study outlines a technical workflow and procedures for collecting these types of data, providing a template which can be easily adapted to collect data on vector breeding sites in other ecological contexts. This allows the identification of associations between landscape characteristics and vector breeding sites while also developing training data for deep-learning algorithms.

A key finding of this project was that the development of an environmentally and spatially representative sampling design from freely available satellite data is a feasible method for initial surveys of a landscape for a rare phenomenon when supplemented with local knowledge of the environment and a follow-up stage of intensive sampling. Larval surveys are a common form of entomological sampling; however, their sampling frames can often be designed with convenience or prior knowledge in mind. For example, a habitat type which is known to be associated with a certain vector may be oversampled, leading to biases in the representation of land types present. In the case of the breeding ecology of *An. funestus*, for which there is a paucity of knowledge, it is especially important to sample the full range of habitats present in the study area, to gain knowledge of the areas where aquatic habitats are present but also where they are absent. It is also crucial that a sampling frame will pick up the species of interest and adjust the design to ensure this if necessary. In our study, it is clear, from the low number of potential habitats picked up through the random stratification alone, that this sampling frame required an extension in order to be confident that we could locate rare *An. funestus* aquatic habitats [13, 49]. We extended the sampling frame to account for the rarity of these habitats by searching in the terrain surrounding the randomly selected hexagons, aided by local knowledge of water body distribution. The targeted surveys were a successful method of locating water bodies, leading to 107 potential larval habitats, often in areas which would not have been visited based on convenience sampling. In the case of *An. funestus* breeding habitats in Côte d'Ivoire, we found that this sampling framework served as a strong initial survey which allowed us to locate potential breeding sites. When followed up with an adaptive sampling stage of intensive larval surveys, we were successful in identifying a total of nine *An. funestus* breeding sites.

In this study, we stratified the landscape using Sentinel-2 multispectral bands and vegetation indices which were derived from them. Other sources of freely available satellite remote sensing and spatially explicit data, such as soil type, hydrological indices, and weather reanalysis and forecasting, could have been used for the same stratification purposes and could have produced different numbers and locations of strata. Nonetheless, to validate the usage of Sentinel-2-derived variables to differentiate the landscapes of interest for *An. funestus* breeding, we collected ground-truthing land cover data and performed a sensitivity analysis. Future studies could build on existing data to iteratively develop sampling frames including all key metrics associated with the vector of interest.

The nine *An. funestus* larval habitats found were large, semipermanent water bodies, which made up a small subset of our total sampled habitats. These findings support previous descriptions of *An. funestus* habitats as “few, fixed, and findable” [13, 49]. The cross-sectional nature of our study, however, did not allow us to confirm whether these water bodies are breeding sites consistently over time, where female *An. funestus* returns every breeding season. To investigate this, the same sampling points could be revisited and sampled for *An. funestus* larval presence in the following rainy season. The results of our larval survey could be used in future studies to describe the distribution of *An. funestus* breeding sites within this region.

We trialled a novel protocol for the collection of eDNA samples from potential breeding sites to show the presence of *An. funestus* in water bodies where their larvae were not collected or identified. The previous detection of eDNA from artificial *Anopheles* larval habitats has demonstrated the ability to detect 0.002 larvae/ml, and further validation of our protocol with field samples is ongoing [50]. The SOPs provided and instructions on these methods should serve as good guidelines for future researchers.

One of the key outputs of this study is the development of a workflow using open-source tools to collect and label imagery. We found that the use of offline maps loaded to the ODK forms was essential for efficient navigation in the field and the collection of drone data. The inclusion of gridded guidelines for drone surveys and previously sampled water bodies allowed for efficient mapping of the sampled sites and their surrounding landscapes in real-time. In addition, the online platform Groundwork for categorising our drone imagery provided a user-friendly, collaborative option for the often tedious task of labelling imagery for training datasets for deep-learning methods. The platform enabled good project management with the ability to oversee labellers' progress, and all labelled images were verified by experts who are familiar with the environment. This is an efficient tool for building a catalogue of training data for wider projects using remote-sensing data with the aim of landscape classification using deep-learning methods. Using a landscape classification system which is based on the literature of *An. funestus* breeding ecology should ensure its applicability for further research into land cover risk factors for the vector's larval habitats and predictive mapping based on land cover.

Another important consideration in projects involving drone mapping and entomological sampling is the seasonal changes which may occur during the project. This is especially important in rapidly changing environments, for example, in an area undergoing deforestation events, and during changing seasons, for example, in the transition months between dry and rainy seasons. These biotic and abiotic changes can have significant impacts on mosquito ecology [51] and can also be visible in remote-sensing imagery. One way to adjust a drone and entomology protocol to account for this is to aim to collect drone and entomology data simultaneously. Our protocol aimed to map a potential larval habitat no more than two days after sampling, to ensure that the images collected reflected the characteristics of the water body at the time of sampling. However, due to logistical issues, there was a lag of over two weeks when mapping some water bodies. The extent of the area to be mapped also resulted in a timespan of six weeks between the first and the last drone flights. In the case of this study, we do not expect these time lags to have a major impact on the imagery analysis, as the rainy season which was due to commence during our data collection period arrived late, and the weather remained relatively stable throughout. We do, however, recommend taking time lags into account when planning drone and entomological surveys.

This project was somewhat limited by technical issues encountered with drones. On two occasions, our drones lost connection with the remote controller while in flight, resulting in irreparable damage. Although there were drone merchants in Cote d'Ivoire, procurement of new drones was slow for the short time span of this project. The original protocol for this study aimed to map a 3 km buffer around the study villages; however, due to technical issues this was not feasible within the study time frame. The protocol changed to reflect this, and we focused mapping efforts on sampled water bodies and the landscape immediately surrounding them, rather than mapping the full region of interest. Technical issues are an important factor to take into account when planning a project which involves drone mapping, and we recommend adding contingency time and budget to any fieldwork planning. We strongly suggest always using the equipment according to the manufacturer's instructions and taking special caution to keep equipment from overheating, especially in sunnier environments. It is useful to establish in-country connections with drone vendors prior to beginning fieldwork, and if possible, to have backup equipment should any technical issues arise.

## 6. Conclusions

This paper provides a technical workflow on the development of an environmentally and spatially representative sampling design using remote-sensing data and the collection of drone imagery and larval distribution data for deep-learning methods. We hope that it can act as a useful guide for the integration of drone and vector data collection for future research. We found that using a randomly stratified sampling design which is representative of the landscape supplemented by local knowledge, and a second stage of intensive sampling, serves as a strong framework for identifying the “few, fixed, and findable” breeding sites of *An. funestus* in our study villages.

## Figures and Tables

**Table 1:** Physicochemical characteristics of interest to be collected in each water body.

Variable	Equipment and collection method
Location	Tablet ODK GPS
Type of water body	Observation
Size of water body	Approximation, with larger water bodies verified using drone imagery
Proportion of emergent vegetation cover	Approximation to the nearest 10%
Predominant type of emergent vegetation present	Observation, as classified in [40]
Canopy cover	Approximation to the nearest 10%
Water transparency	Observation against a white background (dipper)
Water permanence	Observation
Water movement	Observation
Water depth	Ruler
Total dissolved solids	Total dissolved solids measurement device

**Table 2:** Land cover and water body types associated with *An. funestus* larval and adult presence.

Reference	Location	Land cover type						Type of water body
		Agriculture	Human settlement	River	Savannah	Swamp	Others	
[41]	Benin	Small scale			Yes			—
[32]	Cameroon	Yes		Yes				Small water bodies, cattle footprints
[42]	Cameroon	Cropland			Yes		Deciduous woodland	—
[43]	Malawi	Small scale					Shrub and grassland	—
[18]	Kenya, Tanzania		Yes	Yes		Yes		Swamps, vegetated (grass) stream and river edges, and vegetated pools
[10]	Kenya	—	Large, semipermanent with aquatic vegetation and algae					
[28]	Tanzania			Yes				Ephemeral river channels
[11]	Kenya						Highlands	Streams, drainage ditch habitats
[27]	Kenya	—	Shaded water					
[12]	Ethiopia	Irrigated landscape						Puddles of lowland dam, and irrigation canals
[21, 22]	Kenya					Yes	Grass cover	Swamps, drainage ditches, and abandoned gold mines
[19]	Mozambique					Yes		Swamps with reed grass
[17, 44]	Kenya					Yes	Pasture	Swamps and permanent water bodies
[45]	Kenya	—	Stream pools					
[13]	Tanzania	Rice and maize	Yes	Yes				Small spring-fed pools, medium-sized semipermanent natural ponds, slow-moving water along river tributaries
[20]	Kenya	Yes				Cultivated		—
[46]	Zimbabwe	Small-scale farming along rivers (maize, bananas, and yams)		Yes	Tropical savannah		Sparse woodland	Irrigation canals, marshes, and shallow wells
[47]	Eritrea		Yes					Streams
[48]	Nigeria	Farmland	Yes					Nonmoving water bodies, cemented reservoirs, rice farms, ponds, pools, and overhead tanks

**Table 3:** The number of hexagonal strata and their proportions sampled according to a randomly stratified sampling design, with the number of each being positive for water bodies.

Strata	Number of hexagons	Proportional number sampled	Number of sampled hexagons with water bodies
1	1962	41 (2.09%)	0 (0.00%)
2	1545	32 (2.07%)	0 (0.00%)
3	1040	22 (2.12%)	1 (4.50%)
4	1231	26 (2.11%)	2 (7.69%)
Total	5778	121 (2.09%)	3 (2.48%)

**Table 4:** The total number of water bodies, and those *Anopheles*-positive habitats, found per sampling strategy.

Sampling strategy	Number of water bodies found	Number of water bodies positive for anopheline larvae
Randomly stratified	3	0 (0%)
Targeted	107	62 (72%)
Adaptive	79	57 (59%)

**Table 5:** Summary statistics for the characteristics of anopheline larval positive habitats.

Variable		Positive sites (range)	Negative sites (range)
Average canopy cover (%)		37 (0, 100)	28 (0, 100)
Average emergent vegetation cover (%)		51 (0, 100)	27.2 (0, 100)
Average total dissolved solids (ppm)		75 (15, 216)	97 (5, 240)
Proportion of potential habitats positive by water permanence	Temporary	0.47	0.53
	Semipermanent	0.61	0.66
	Permanent	0.61	0.33
Proportion of potential habitats positive by water flow	Stationary	0.67	0.33
	Slow flowing	0.25	0.75
	Fast flowing	0.00	0.00
Proportion of potential habitats positive by water turbidity	Transparent	0.67	0.33
	Cloudy	0.65	0.35
	Very cloudy	0.65	0.35
	Opaque	0.67	0.33

## Data Availability

The data that support the findings of this study can be obtained from the corresponding author upon reasonable request.

## Supplementary Materials

Supplementary Materials (1) Google Earth Engine script for preprocessing of Sentinel-2 imagery and computation of vegetation indices. (2) Standard Operating Procedures (SOPs) for (a) Larval surveys, rearing, and species identification; (b) environmental DNA collection and processing; and (c) drone mapping with DJI Phantom 4. (3) Field equipment lists for (a) larval, water body, eDNA, and ground-truth surveys and (b) drone surveys. (4) Drone flight log. (5) Drone information sheet in English. ()
