# Supplementary material for: Technical Workflow Development for Integrating Drone Surveys and Entomological Sampling to Characterise Aquatic Larval Habitats of Anopheles funestus in Agricultural Landscapes in Côte d'Ivoire
Source: J Environ Public Health. 2021 Nov 1;2021:3220244. doi: 10.1155/2021/3220244 (PMC8575637; doi:10.1155/2021/3220244)

**Supplementary Material**

1. **Google Earth Engine script for pre-processing of Sentinel-2 imagery and computation of vegetation indices**

<https://code.earthengine.google.com/f4fc0e304d1ffa10a01204f9329872cf>

1. **Standard Operating Procedures (SOP)**

***2A. Larval surveys, rearing and species identification***

Larval collections:

1. Conduct ten dips at the perimeter or near the vegetation of each water body

2. Avoid disturbing or casting a shadow over the water body during dipping

3. Immerse dipper in water body at 45-degree angle 2 cm below the water surface

4. Allow the dipper to fill ¾ with water and withdraw quickly but gently

5. Allow 3 minutes interval between dips to allow larvae to return to surface

6. If water covered with vegetation or organic debris, agitate water to cause larvae to sink, clear vegetation, wait 3 minutes for larvae to return to surface then dip

7. Count the number of anopheline L1/L2, L3/L4 larvae and pupae in all the dips

8. Use a pipette to transfer all larvae from the dipper to a plastic vial labelled with a QR code (which specifies the village, strata, hexagon number and water body ID)

Larval rearing and identification:

1. Bring vials to Institut Pierre Richet entomology lab

2. Transfer contents of vials into labelled plastic containers covered with mesh secured with elastic bands

3. Add 400ml of ambient temperature distilled water to plastic container

4. Transfer pupae into plastic cups inside cages

5. When adults emerge from water, extract using aspirator and kill by freezing

6. Identify adult species morphologically

7. For *An. gambiae and An. funestus* sensu lato adults, confirm species complex identification by PCR

***2B. Environmental DNA collection and processing***

eDNA collection:

1. Conduct one dip at the point of larval collection

2. Add ~40 ml into a 50 ml Falcon tube

3. Keep samples in a cooler with ice during transport to entomology lab

4. Store samples in a -80ºC freezer (alternatively, -20ºC overnight until -80ºC freezer is available)

eDNA extraction:

1. Add 15 ml of collected water to a sterile 50 ml Falcon tube and immediately add 1.5 ml of 3M sodium acetate (pH 5.2), followed by 33 ml of absolute ethanol

2. Store samples overnight at -20ºC

3. Centrifuge samples at 8000 rpm at 6ºC for 30 minutes

4. Discard supernatant

5. Wash pellet in 20 ml of absolute ethanol by centrifuging at 8000 rpm at 6ºC for 10 minutes

6. Discard supernatant

7. Evaporate remaining ethanol at 65ºC

8. Dissolve pellets in 720 uL ATL buffer and 80 uL proteinase K (part of the Qiagen DNeasy 96 blood and tissue kit, Qiagen UK)

9. Incubate samples at 56ºC overnight

10.  Follow remaining Qiagen DNeasy 96 blood and tissue kit protocol

11. Store extracted samples at -20ºC for downstream PCR analysis

***2C. Drone mapping with DJI Phantom 4***

| (1) Before leaving the office | | |
| --- | --- | --- |
|  | Check the weather forecast: there should be no rain and low wind speeds of under 10 m/s | |
|  | Batteries charged (drone, remote control, portable chargers, computer) | |
|  | Drone and remote control (RC) firmware are fully updated. To update, connect the phone/tablet to the RC. Switch on the RC and the drone and launch the DJI GO 4 application. If an update is required, a notification specifying “new firmware” will show. With internet connection, follow on-screen instructions to update. | |
|  | Memory card | |
|  | Local regulations | |
|  | Download offline maps onto pix4dcapture and DJI GS Pro applications | |
|  | On QGIS, create grids of standard sizes (e.g. 750x750 m) over area of interest and print for reference | |
| (2) In the field, set up flight | | |
|  | Check the weather. Wind speed should not be over 10 m/s and the drone cannot fly in rain, snow or fog. If the wind conditions are difficult to judge, conduct a test flight and bring the drone back if winds exceed 12 m/s. | |
|  | Perform general inspection | |
|  |  | Check drone body and propellers for damage |
|  |  | Confirm the sensors are not blocked and camera lens is clean |
|  | Assemble RC | |
|  |  | Connect the iPad/iPhone/android to the RC (using USB cable) |
|  |  | Unfold RC antennas so they are flat and fully extended |
|  |  | Turn on the control by pressing twice: a quick press following a long press (a sound should indicate the RC is turned on). |
|  |  | Use the small slider on the right side of the RC to set the mode to P (positioning).  *P-mode works best when the GPS signal is strong. The drone uses the GPS and Vision Systems to locate itself, stabilize, and navigate between obstacles. Intelligent Flight Modes are enabled in this mode. The maximum speed is 50 kph. S-mode disables the Vision Systems and only uses the GPS for positioning. The drone cannot sense or avoid obstacles. The maximum speed is 72 kph. T-mode is based on P-mode and limits the flight speed to make the drone more stable during shooting. Intelligent Flight Modes are not available in this mode. The maximum speed, maximum ascend speed, and maximum descend speed are 1 m/s.* |
|  | Assemble drone | |
|  |  | Attach propellers by pushing down gently and turning the motor in the direction indicated on each individual propeller. Propellers with silver circular marks should be attached to the motors with silver marks. This applies to propellers with black circular marks too. Check propellers are attached properly by spinning them manually. |
|  |  | Remove any camera coverings |
|  |  | Make sure there is space available on SD card |
|  |  | Place the drone flat on the ground in the location where it will be launched from |
|  |  | Insert the battery into the battery compartment. Turn on the battery by pressing twice: a quick press following a long press (a sound should indicate the drone is turned on). Automatic checks will be performed after the battery is turned on. |
|  |  | Calibrate the compass if needed.  *The compass should be calibrated when flying outdoors when: 1) flying at a location farther than 50km away from the location the drone was last flown; 2) the drone has not been flown for more than 30 days; 3) the compass interference warning appears on the DJI GO 4 app and/or the Aircraft Status Indicators blink alternating red and yellow.*  *To calibrate, tape the System Status Bar in DJI Go 4 and select “Calibrate.” Follow the on-screen instructions. Hold the drone horizontally and rotate it 360 degrees. The Indicator lights will turn solid green. Hold the drone vertically with the nose pointing downward and rotate 360 degrees along the vertical axis. The Indicator lights will blink red if the calibration has failed. Change the location and try the calibration process again.* |
|  | A second person to cross-check that drone and RC has been assembled correctly | |
|  | Plan flight | |
|  |  | Open the Pix4Dcapture application. Select Grid Mission. |
|  |  | Tap the positioning icon (target icon) on the bottom left hand side to center the area around you |
|  |  | Tap the new mission icon (counterclockwise arrow) to create a new mission grid |
|  |  | Select the area to be mapped. The red points of the drone on the app match up with the red lights on the drone. The same applies to the green points. Check that the grid on Pix4D aligns with the grid on the drone map in the ODK software on tablets. |
|  |  | On the left-hand side, adjust the height to the desired altitude (maximum 150m). |
|  |  | Tap the gear icon at the top to launch the settings menu. Select speed, camera angle (vertical), overlap (minimum 80%), and face (forward). |
|  |  | Tap the floppy disc save icon on the bottom right corner. |
|  |  | Record the flight details in the Drone Log spreadsheet, including date, location, camera used and name the flight with the date and consecutive number i.e. YYYYMMDD_## |
| (3) Fly the drone | | |
|  | Tap and hold the green “Start” icon on the Pix4Dcapture app to begin the flight. The drone will take-off, conduct the flight, and land on its own. | |
|  | In sunny conditions, keep the iPad/iPhone shaded under an umbrella to prevent overheating | |
|  | Monitor the progress of the drone and the wind speed and battery level.  *If the wind speed exceeds 10 m/s or the battery gets too low (depending on distance from the landing site), bring the drone back by tapping the “Abort” button.* | |
|  | The propellers should stop automatically if auto landing is used.  *If they do not, there are two methods to stop the motors: (1) Push and hold the left joystick down. The motors will stop after three seconds or (2) Push both joysticks to the inner OR outer bottom corners and hold until the motors have stopped (this is the same method to start the motors).* | |
|  | Do not turn off the controller, drone, or app until the pictures finish downloading. | |
|  | Record any issues observed with the flight in the Drone Log | |
| (4) After flight | | |
|  | Remove battery and disassemble | |
| (5) Download data | | |
|  | The pictures and flight details are saved to the phone/tablet used. Plug in the phone/tablet to a computer and locate the phone/tablet’s files | |
|  | Open the Pix4D folder. The flights are organized in chronological order and called “Missions ##.” Open the required mission folder and then open the “Data” folder to see the images. | |
|  | Give the flight the same name as in the Drone Log. | |
|  | Save the pictures where desired. Delete the folder from the phone/tablet after confirming it has been copied properly. | |
|  | If not using a SD card, delete the images from the drone. Turn on the drone and controller and connect the phone/tablet to the DJI GO 4 app. On the main screen, tap the media icon (triangle) in the bottom left-hand corner. Select the images (grouped together by date) and tap “Delete”. | |
|  | Record that the file has been downloaded in the drone log | |
| (6) Image processing | | |
|  | To check data quality, do a rapid process and report | |
|  |  | Open the Pix4D file created |
|  |  | Make sure initial project processing is the only step selected |
|  |  | Select rapid processing |
|  |  | Check the report for image quality and coverage |
|  | To fully process the image | |
|  |  | Open the Pix4D file created |
|  |  | Select all steps and full processing. Processing will take 2-3 hours |
|  |  | Record the image has been processed in the drone log |
| (7) Data management | | |
|  | File directories will be automatically generated during processing. Make sure all directories are named correctly and can be linked to the drone log | |
| (8) Battery storage | | |
|  | All batteries must be stored at room temperature partially charged. Keep in a protective bag and do not over charge.The battery automatically discharges to less than 70% of the maximum battery level when it is idle for more than 10 days. It takes approximately three-four days to discharge the battery to 60%. | |

**3. Field equipment**

***3A. Larval, water body, eDNA and ground-truth survey***

| **Mosquito larvae** | **Water parameters** | **eDNA** | **General** |
| --- | --- | --- | --- |
| Dippers 350ml x4 | Ruler | Falcon tubes 50ml x300 | ODK tablet x2 |
| White dishes x5 | Total dissolved solids meter | Freezer 10L | Portable chargers x2 |
| Vials x100 |  | Ice packs | Masking tape + marker |
| Pipettes x20 |  |  | GeoTrace ODK form |
| QR code labels |  |  | Wellingtons |

***3B. Drone survey***

| **DJI Phantom 4 Pro** | **DJI Mavic 2 Pro** | **General** |
| --- | --- | --- |
| Batteries x5 | Batteries x3 | SD cards + USB reader |
| Charging hub + charger x2 | Charging hub + charger x2 | SD card reader |
| Remote control | Remote control | Laptop + external hard drive |
| iPad (Pix4Dcapture/DJI GS Pro) + cable | iPhone/android (Pix4Dcapture) + cable | Glue + tape + rubber bands |
| Propellers + spares | Propellers + spares | Aluminium foil |
|  | Parrot sequoia and mount | Portable chargers x2 |
|  |  | Information sheets |
|  |  | Flight log |
|  |  | Offline maps downloaded |
|  |  | Extension cord |
|  |  | Umbrella |

**4. Drone flight log**

| **Date** | **Location** | **Drone** | **Camera**  1) RGB | **Clouds**  1) Clear  2) Overcast  3) Partly cloudy | **Wind condition**  1) Calm  2) Medium  3) High | **Notes** | **Flight ID** |
| --- | --- | --- | --- | --- | --- | --- | --- |
|  |  |  |  |  |  |  |  |
|  |  |  |  |  |  |  |  |

**5. Drone information sheet in English**


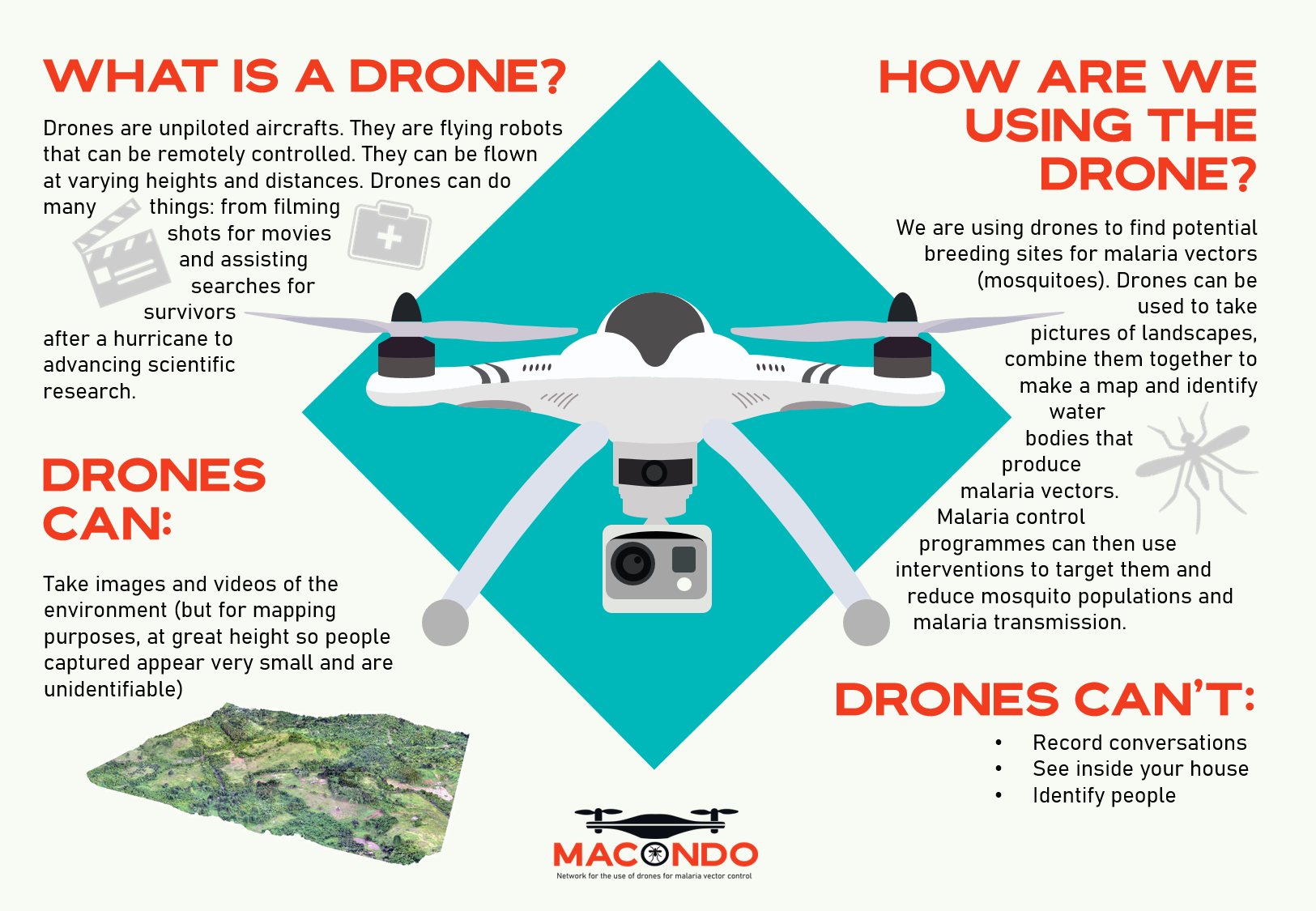

Supplement: Supplementary Materials — (1) Google Earth Engine script for preprocessing of Sentinel-2 imagery and computation of vegetation indices. (2) Standard Operating Procedures (SOPs) for (a) Larval surveys, rearing, and species identification; (b) environmental DNA collection and processing; and (c) drone mapping with DJI Phantom 4. (3) Field equipment lists for (a) larval, water body, eDNA, and ground-truth surveys and (b) drone surveys. (4) Drone flight log. (5) Drone information sheet in English. () [file 3220244.f1.docx]
